# Detecting myocardial infarction in critical illness using screening troponin measurements and ECG recordings

**DOI:** 10.1186/cc6815

**Published:** 2008-03-04

**Authors:** Wendy Lim, Paula Holinski, PJ Devereaux, Andrea Tkaczyk, Ellen McDonald, France Clarke, Ismael Qushmaq, Irene Terrenato, Holger Schunemann, Mark Crowther, Deborah Cook

**Affiliations:** 1 Department of Medicine, McMaster University, Canada; 2 Department of Clinical Epidemiology and Biostatistics, McMaster University, Canada; 3 Department of Medicine, King Faisal Specialist Hospital and Research Center, Jeddah, Saudi Arabia; 4 Department of Epidemiology, Italian National Cancer Institute Regina Elena, Rome, Italy

## Abstract

**Introduction:**

To use screening cardiac troponin (cTn) measurements and electrocardiograms (ECGs) to determine the incidence of elevated cTn and of myocardial infarction (MI) in patients admitted to the intensive care unit (ICU), and to assess whether these findings influence prognosis. This is a prospective screening study.

**Materials and methods:**

We enrolled consecutive patients admitted to a general medical-surgical ICU over two months. All patients underwent systematic screening with cTn measurements and ECGs on ICU admission, then daily for the first week in ICU, alternate days for up to one month and weekly thereafter until ICU death or discharge, for a maximum of two months. Patients without these investigations ordered during routine clinical care underwent screening for study purposes but these results were unavailable to the ICU team. After the study, all ECGs were interpreted independently in duplicate for ischaemic changes meeting ESC/ACC criteria supporting a diagnosis of MI. Patients were classified as having MI (elevated cTn and ECG evidence supporting diagnosis of MI), elevated cTn only (no ECG evidence supporting diagnosis of MI), or no cTn elevation.

**Results:**

One hundred and three patients were admitted to the ICU on 112 occasions. Overall, 37 patients (35.9 per cent) had an MI, 15 patients (14.6 per cent) had an elevated cTn only and 51 patients (49.5 per cent) had no cTn elevation. Patients with MI had longer duration of mechanical ventilation (p < 0.0001), longer ICU stay (p = 0.001), higher ICU mortality (p < 0.0001) and higher hospital mortality (p < 0.0001) compared with those with no cTn elevation. Patients with elevated cTn had higher hospital mortality (p = 0.001) than patients without cTn elevation. Elevated cTn was associated with increased hospital mortality (odds ratio 27.3, 95 per cent CI 1.7 – 449.4), after adjusting for APACHE II score, MI and advanced life support. The ICU team diagnosed 18 patients (17.5 per cent) as having MI on clinical grounds; four of these patients did not have MI by adjudication. Thus, screening detected an additional 23 MIs not diagnosed in practice, reflecting 62.2 per cent of MIs ultimately diagnosed. Patients with MI diagnosed by the ICU team had similar outcomes to patients with MI detected by screening alone.

**Conclusion:**

Systematic screening detected elevated cTn measurements and MI in more patients than were found in routine practice. Elevated cTn was an independent predictor of hospital mortality. Further research is needed to evaluate whether screening and subsequent treatment of these patients reduces mortality.

## Introduction

Diagnosing myocardial infarction in critically ill patients is challenging [1]. Ischaemic chest pain is uncommon due to analgesic use and communication of ischaemic symptoms – when they occur – is hampered, since these patients are frequently endotracheally intubated, sedated or comatose. The second challenge in MI diagnosis is that cTn levels are typically only measured in critically ill patients with known coronary artery disease, or when MI is considered as an explanation for hypotension or arrhythmia. Thus, it is possible that many elevated cTn levels are never identified and as a result, that the diagnosis of MI in the intensive care unit (ICU) is frequently missed [2]. Furthermore, interpretation of an elevated cTn level in the ICU is challenging because conditions other than MI which cause cTn elevation [3,4] (such as pulmonary embolism) occur in critically ill patients [5]. For this reason, diagnosis of MI requires criteria in addition to elevated cTn levels such as electrocardiogram (ECG) evidence of myocardial ischaemia [6].

In a meta-analysis of 23 studies involving 4,492 critically ill patients, elevated cTn was associated with an adjusted odds ratio (OR) for death of 2.5 (95 per cent confidence interval [CI] 1.9–3.4) and an increased mean length of ICU stay of three days (95% CI 0.98–5.05) [7]. This suggests that an elevated cTn measurement in the ICU setting has prognostic relevance. However, many studies in this meta-analysis did not undertake screening cTn measurements to detect clinically silent MIs. The objective of this study was to use screening cTn measurements and ECGs to determine the incidence of elevated cTn and MI in consecutive patients admitted to the ICU, and to assess whether these findings influence prognosis.

## Materials and methods

### Overview

We screened all consecutive patients admitted to the ICU at St. Joseph's Hospital, Hamilton, Ontario, Canada from 2 January 2006 to 1 March 2006. On ICU admission, we collected patient demographics (age, sex, admitting diagnosis, Acute Physiology and Chronic Health Evaluation [APACHE] II score) and baseline data (pre-existing medical conditions, cardiac risk factors and medications).

All cTn and ECGs ordered by the ICU team were collected. We obtained screening cTn and ECGs if not ordered by the ICU team; for these screening tests, we obtained deferred consent from a family member as soon as possible after ICU admission. If consent was declined for screening cTn and ECGs, we retained the data that the ICU team ordered, including cTn and ECGs. Our institutional Research Ethics Board approved this study.

### Setting

The ICU at St Joseph's Hospital is a 15-bed, university-affiliated medical-surgical ICU. Although the hospital has a coronary care unit for patients with primary cardiac diagnoses or requiring telemetry, such patients also requiring mechanical ventilation and those receiving inotropes and/or vasopressors are admitted to the ICU. The ICU is a closed unit staffed by intensivists and physicians-in-training.

### Study participants

All consecutive patients admitted to the ICU during the recruitment period were enrolled. There were no exclusion criteria.

### Screening investigations

#### Troponin measurements

If the ICU team ordered cardiac troponin T (cTnT) for clinical purposes, a trained Research Coordinator recorded these values. If the ICU team did not order cTnT, blood was collected for measurement of cTnT. cTnT measurement was performed at ICU admission, then daily for the first week in ICU, followed by alternate days for up to one month and then weekly thereafter until ICU death or discharge, for a maximum of two months.

All cTnT assays were run in real-time. The ICU team had access to the cTnT results that they ordered through the hospital computer laboratory system, as per usual practice. To ensure that levels drawn for study purposes would not influence patient management, laboratory personnel entered screening test results in a password protected computer system and results were not accessible to the ICU team.

Blood samples for cTnT measurements were drawn into EDTA tubes, and plasma for sample analysis obtained following centrifugation of whole blood at 1,500 g for 15 minutes. cTnT was measured using an electrochemiluminescence immunoassay (Roche Modular analytics E170 [Elecsys module] immunoassay analyser, Roche Diagnostics, Indianapolis, IN). The analytical sensitivity (lower detection limit) of this assay is 0.01 μg/L. An elevated cTn was defined as values greater than or equal to 0.04 μg/L, which represents the assay coefficient of variation of 10 per cent.

#### Electrocardiography

Any 12-lead ECGs (PageWriter, Hewlett-Packard, Palo Alto, CA) ordered by the ICU team for clinical purposes were photocopied by the Research Coordinator. If the ICU team did not order ECGs, the Research Coordinator performed screening ECGs on ICU admission, then daily for the first week in ICU, followed by alternate days for the remainder of the first month and then weekly thereafter until ICU death or discharge, for a maximum of two months. To ensure that ECGs done for study purposes would not influence patient management, screening ECGs were printed out and immediately placed in the research chart so the results were not accessible to the ICU team. These ECGs were not reviewed by research staff until after the study was complete; they were never made available to the ICU team.

At study completion, two investigators (IQ, DJC) interpreted ECGs in duplicate, blinded to each others' assessments and to all clinical information with the exception of cTn levels. All ECGs were adjudicated for the presence of ischaemic changes supporting ESC/ACC criteria for MI [6]. ECGs adjudicated as having ischaemic changes consistent with MI were then further classified as ST or non-ST elevation MI (STEMI or NSTEMI, respectively). All patient identifiers were removed from the ECGs prior to interpretation. To replicate clinical practice, the computer-generated ECG interpretation printed on the ECGs was not removed. Discrepancies were resolved by discussion.

### Data collection

On ICU admission, we collected patient demographics and baseline data. Daily, we collected laboratory results, cardiac medications and anticoagulants, new clinical events and complications that the ICU team detected (development of arrhythmias, pulmonary oedema, non-fatal cardiac arrest and cardiogenic shock), need for advanced life support (mechanical ventilation, inotropes and/or vasopressors, and haemodialysis) and if ischaemic cardiac symptoms were present. We also collected information on whether the ICU team made a diagnosis of MI during the patient's ICU stay.

We prospectively followed all patients throughout their ICU stay, and recorded their vital status and duration of stay in ICU and hospital. Data were collected from the patient's medical chart, the ICU computerised clinical information system, and the hospital laboratory system. Data were recorded on paper case report forms and entered into an Excel program for analysis.

### Outcomes

#### Outcome definitions

We defined an elevated cTn as one or more measurements of cTnT with values greater than or equal to 0.04 μg/L. We defined MI as one or more measurements of elevated cTn and one or more ECGs adjudicated as having ischaemic changes meeting ESC/ACC criteria supporting a diagnosis of MI; the elevated cTn and ECG changes had to be contemporaneous to establish a diagnosis of MI.

#### Patient classification

We classified patients into three groups: MI (as defined above); elevated cTn only (one or more measurements of elevated cTn during ICU admission and all ECGs classified as having no ischemic changes supporting ESC/ACC criteria for MI); or no cTn elevation.

### Statistical analysis

We report continuous data as mean and standard deviation or median and interquartile range, as appropriate. Binary data are reported as frequency and percentage values. We compared continuous variables using the Kruskall-Wallis non-parametric test and the Mann-Whitney paired test to explore the differences among groups, and categorical variables using the Pearson's Chi-square test or Fisher's exact test, when appropriate. Due to multiple significance testing, a p value < 0.01 was considered statistically significant. In the multivariable regression analysis, we adjusted for APACHE II score and advanced life support (mechanical ventilation, inotropes and/or vasopressors, and haemodialysis at any time in the ICU), to examine the association between elevated cTn and MI and ICU and hospital mortality. A sensitivity analysis was performed whereby inotropes and/or vasopressors were not included as covariates in the regression analysis, since MI may result in inotrope and/or vasopressor dependence, which may attenuate the association between an elevated cTn/MI and mortality. We express associations using odds ratios (ORs) and 95 per cent CIs.

## Results

Over the two-month study period, there were 112 ICU admissions representing 103 unique patients (nine patients were admitted twice). We obtained deferred consent for 89 patients (86.4 per cent). No consent was obtained for fourteen patients (13.6 per cent), and no screening cTn and ECG data were collected on these patients; however, all data available in the medical record and any cTn and ECGs that the ICU team ordered for clinical care were recorded. Nine of these 14 patients either died shortly after ICU admission (due to severe illness or withdrawal of life support), or had short ICU admissions (such as being admitted for airway protection) and cTn and ECGs were typically ordered by the ICU team for clinical care so there were few missed screening tests.

The clinical characteristics of the 103 enrolled patients are shown in Table 1. Most patients were medical (63.1 per cent) and the admitting APACHE II diagnoses included: cardiovascular (13.8 per cent), respiratory (21.6 per cent), gastrointestinal (12.3 per cent), neurologic (9.3 per cent), sepsis (13.8 per cent), metabolic (16.9 per cent) and haematologic (12.3 per cent). Only one of the nine patients admitted with a cardiovascular diagnosis had an admission diagnosis of acute MI. The remaining 36.9 per cent were surgical patients, with 27 (71.1 per cent) undergoing elective surgery and 11 (28.9 per cent) undergoing emergent surgery.

**Table 1:** Clinical characteristics of enrolled patients

	Total	MI	Elevated cTn only	No cTn elevation	p value^3^	p value^4^
			
	(*N *= 103)	(*N *= 37)	(*N *= 15)	(*N *= 51)		
Age, mean (SD)	64.1 ± 17.5	69.9 ± 14.9	64.3 ± 21.8	59.7 ± 16.9	0.018	- MI vs cTn, p = 0.467 - cTn vs no cTn elevation, p = 0.309 - MI vs no cTn elevation, p = 0.004
Female sex, N (%)	44 (42.7)	16 (43.2)	4 (26.7)	24 (47.1)	0.372	- MI vs cTn, p = 0.352 - cTn vs no cTn elevation, p = 0.236 - MI vs no cTn elevation, p = 0.829
APACHE II score, mean (SD)	21.4 ± 10.3	28.2 ± 9.5	22 ± 8.3	16.2 ± 8.3	<0.0001	- MI vs cTn, p = 0.045 - cTn vs no cTn elevation, p = 0.014 - MI vs no cTn elevation p < 0.0001
Medical, N (%)	65 (63)	32 (86.5)	10 (66.7)	23 (45.1)	<0.0001	- MI vs cTn, p = 0.129 - cTn vs no cTn Elevation p = 0.240 - MI vs no cTn elevation p < 0.0001
Past medical history, N (%)						
Smoking	28 (27.2)	10 (27.0)	4 (26.7)	14 (27.5)	0.998	- MI vs cTn, p = 1.000 - cTn vs no cTn elevation, p = 1.000 - MI vs no cTn elevation, p = 1.000
Hypertension	54 (52.4)	24 (64.9)	9 (60.0)	21 (41.2)	0.073	- MI vs cTn, p = 0.760 - cTn vs no cTn elevation, p = 0.245 - MI vs no cTn elevation, p = 0.033
Diabetes mellitus^1^	25 (24.3)	13 (35.1)	4 (26.7)	8 (15.7)	0.107	- MI vs cTn, p = 0.747 - cTn vs no cTn elevation, p = 0.446 - MI vs no cTn elevation, p = 0.044
Hyperlipidemia	17 (16.5)	6 (16.2)	5 (33.3)	6 (11.8)	0.141	- MI vs cTn, p = 0.260 - cTn vs no cTn elevation, p = 0.107 - MI vs no cTn elevation, p = 0.549
Documented coronary disease/angina	3 (2.9)	1 (2.7)	1 (6.7)	1 (2.0)	0.632	- MI vs cTn, p = 0.498 - cTn vs no cTn elevation, p = 0.406 - MI vs no cTn elevation, p = 1.000
Prior myocardial infarction	5 (4.9)	4 (10.8)	1 (6.7)	0 (0)	0.062	- MI vs cTn, p = 1.000 - cTn vs no cTn elevation, p = 0.227 - MI vs no cTn elevation, p = 0.028
Congestive heart failure	16 (15.5)	10 (27)	2 (13.3)	4 (7.8)	0.048	- MI vs cTn, p = 0.470 - cTn vs no cTn elevation, p = 0.612 - MI vs no cTn elevation, p = 0.020
Peripheral vascular disease	13 (12.6)	7 (18.9)	3 (20.0)	3 (5.9)	0.124	- MI vs cTn, p = 1.000 - cTn vs no cTn elevation, p = 0.125 - MI vs no cTn elevation, p = 0.088
Stroke/transient ischemic attack	10 (9.7)	5 (13.5)	3 (20.0)	2 (3.9)	0.112	- MI vs cTn, p = 0.676 - cTn vs no cTn elevation, p = 0.073 - MI vs no cTn elevation, p = 0.126
Baseline life support interventions, N (%)						
Ventilation						
Invasive mechanical ventilation	59 (57.3)	27 (73.0)	7 (46.7)	25 (49.0)	0.054	- MI vs cTn, p = 0.108 - cTn vs no cTn elevation, p = 0.100 - MI vs no cTn elevation, p = 0.029
Non-invasive mechanical ventilation	4 (3.9)	1 (2.7)	1 (6.7)	2 (3.9)	0.799	- MI vs cTn, p = 0.498 - cTn vs no cTn elevation, p = 0.545 - MI vs no cTn elevation, p = 1.000
Inotropes and vasopressors, N (%)						
Epinephrine	3 (2.9)	3 (8.1)	0 (0)	0 (0)	0.064	- MI vs cTn, p = 0.548 - cTn vs no cTn elevation, p = NE - MI vs no cTn elevation, p = 0.071
Dopamine^2^	6 (5.8)	5 (13.5)	0 (0)	1 (2.0)	0.043	- MI vs cTn, p = 0.305 - cTn vs no cTn elevation, p = 1.000 - MI vs no cTn elevation, p = 0.079
Norepinephrine	14 (13.6)	9 (24.3)	2 (13.3)	3 (5.9)	0.045	- MI vs cTn, p = 0.477 - cTn vs no cTn elevation, p = 0.318 - MI vs no cTn elevation, p = 0.024
Dobutamine	2 (1.9)	2 (5.4)	0 (0)	0 (0)	0.162	- MI vs cTn, p = 1.000 - cTn vs no cTn elevation, p = NE - MI vs no cTn elevation, p = 0.174
Phenylephrine	3 (2.9)	3 (8.1)	0 (0)	0 (0)	0.064	- MI vs cTn, p = 0.548 - cTn vs no cTn elevation, p = NE - MI vs no cTn elevation, p = 0.071
Vasopressin	1 (1.0)	1 (2.7)	0 (0)	0 (0)	0.406	- MI vs cTn, p = 1.000 - cTn vs no cTn elevation, p = NE - MI vs no cTn elevation, p = 0.420
Hemodialysis, N (%)						
Intermittent dialysis	9 (8.7)	6 (16.2)	1 (6.7)	2 (3.9)	0.125	- MI vs cTn, p = 0.658 - cTn vs no cTn elevation, p = 0.545 - MI vs no cTn elevation, p = 0.065
Continuous renal replacement therapy	1 (1.0)	1 (2.7)	0 (0)	0 (0)	0.406	- MI vs cTn, p = 1.000 - cTn vs no cTn elevation, p = NE - MI vs no cTn elevation, p = 0.420

APACHE = Acute Physiology and Chronic Health Evaluation; cTn = cardiac troponin; MI = myocardial infarction; NE = not estimable; ^1^Managed with oral agents and/or insulin; ^2^>3 μg/kg/min; ^3^p value indicates a 3-way comparison; ^4^p value indicates a 2-way comparison.

A total of 37 patients (35.9 per cent) had MI, 15 patients (14.6 per cent) had elevated cTn only (that is isolated cTn elevation), and 51 patients (49.5 per cent) had no cTn elevation. Of the 37 patients with MI, 34 (91.9 per cent) had NSTEMI, and three (8.1 per cent) had STEMI. At baseline, patients with MI were older, more commonly admitted for medical reasons and had higher APACHE II scores than patients without cTn elevation.

Table 2 shows the morbidity and mortality outcomes of the patients with MI, elevated cTn only and no cTn elevation. Patients with MI had longer duration of mechanical ventilation (median 4 versus 1 day, p < 0.0001), increased duration of ICU stay (median 5 versus 2 days, p = 0.001), higher ICU mortality (37.8 per cent versus 2.0 per cent, p < 0.0001) and higher hospital mortality (43.2 per cent versus 2.0 per cent, p < 0.0001) compared to patients with no cTn elevation. Patients with elevated cTn had higher hospital mortality (26.7 per cent versus 2.0 per cent, p = 0.001) compared with patients without cTn elevation. There was no difference in hospital stay among the three patient groups (p = 0.124).

**Table 2:** Frequency of morbidity and mortality outcomes

	MI (*N *= 37)	Elevated cTn only (*N *= 15)	No cTn elevation (*N *= 51)	p value^1^	p value^2^
Duration of mechanical ventilation, median (IQR [days])	4 (0.5–12.5)	2 (0–3)	1 (0–2)	<0.0001	- MI vs cTn, p = 0.022 - cTn vs no cTn elevation, p = 0.167 - MI vs no cTn elevation, p < 0.0001
Duration of ICU stay, median (IQR [days])	5 (2–14)	4 (3–7)	2 (1–4)	0.002	- MI vs cTn, p = 0.336 - cTn vs no cTn elevation, p = 0.026 - MI vs no cTn elevation, p = 0.001
ICU mortality, N (%)	14 (37.8)	1 (6.7)	1 (2.0)	<0.0001	- MI vs cTn, p = 0.020 - cTn vs no cTn elevation, p = 0.357 - MI vs no cTn elevation, p < 0.0001
Duration of hospital stay, median (IQR [days])	15 (4–37)	12 (6–12)	8 (4–19)	0.124	- MI vs cTn, p = 0.785 - cTn vs no cTn elevation, p = 0.175 - MI vs no cTn elevation, p = 0.062
Hospital mortality, N (%)	16 (43.2)	4 (26.7)	1 (2.0)	<0.0001	- MI vs cTn, p = 0.139 - cTn vs no cTn elevation, p = 0.001 - MI vs no cTn elevation, p < 0.0001

cTn = cardiac troponin; ICU = intensive care unit; IQR = interquartile range; MI = myocardial infarction; ^1^p value indicates a three-way comparison; ^2^p value indicates a two-way comparison.

We present predictors of ICU and hospital mortality in Table 3. Both elevated cTn and MI were associated with ICU mortality and hospital mortality in univariable analysis. Elevated cTn was associated with hospital mortality (OR 27.3, 95 per cent CI 1.7–449.4), but not ICU mortality in multivariable analysis after adjusting for illness severity and need for advanced life support. MI was not significantly associated with ICU and hospital mortality in multivariable analysis. Similar results were observed in the sensitivity analysis where inotrope and/or vasopressor use was omitted from the regression; after adjusting for illness severity and need for mechanical ventilation and haemodialysis, elevated cTn was significantly associated with hospital mortality (OR 19.7, 95 per cent CI 1.5–260.4)

**Table 3:** Predictors of ICU and hospital mortality

	ICU mortality		Hospital mortality	
	Univariable	Multivariable	Univariable	Multivariable
	
Predictors	OR (95% CI)	OR (95% CI)	OR (95% CI)	OR (95% CI)

APACHE II score (10-pt increment)	5.7 (2.6–12.6)	3.9 (1.3–11.8)	4.9 (2.5–9.9)	3.5 (1.2–10.1)
Mechanical ventilation	Not estimable^1^	Not estimable^1^	17.3 (2.2–134.8)	7.4 (0.7–79.9)
Inotropes or vasopressors	78.2 (9.6–640.9)	23.6 (2.5–219.3)	17.0 (5.3–54.5)	6.3 (1.3–29.3)
Hemodialysis	2.1 (0.6–7.5)	0.3 (0.04–3.3)	1.4 (0.4–4.8)	0.2 (0.02–2.02)
Elevated cTn	20.3 (2.6–160.4)	2.8 (0.1–70.6)	31.3 (4.0–244.4)	27.3 (1.7–449.4)
MI	19.5 (4.1–92.4)	4.1 (0.3–51.9)	9.3 (3.0–28.5)	0.7 (0.1–4.4)

APACHE = Acute Physiology and Chronic Health Evaluation; CI = confidence interval; cTn = cardiac troponin; ICU = intensive care unit; MI = myocardial infarction; OR = odds ratio; ^1^Not estimable because all patients were either ventilated or died or both.

In terms of complications, most patients did not develop arrhythmias, pulmonary oedema, non-fatal cardiac arrest or cardiogenic shock (Table 4). However, almost twice as many patients with MI developed pulmonary oedema compared with those with no cTn elevation (37.8 per cent versus 11.8 per cent, respectively, p = 0.005).

**Table 4:** Clinical complications

	MI, N (%) (*N *= 37)	Elevated cTn, N (%) (*N *= 15)	No cTn elevation, N (%) (*N *= 51)	p value^1^	p value^2^
Arrhythmia requiring treatment	1 (2.7)	0 (0)	1 (1.8)	0.846	- MI vs cTn, p = 1.000 - cTn vs no cTn elevation, p = 1.000 - MI vs no cTn elevation, p = 1.000
Pulmonary oedema	14 (37.8)	3 (20.0)	6 (11.8)	0.015	- MI vs cTn, p = 0.330 - cTn vs no cTn elevation, p = 0.414 - MI vs no cTn elevation, p = 0.005
Non-fatal cardiac arrest	2 (5.4)	0 (0)	0 (0)	0.162	- MI vs cTn, p = 1.000 - cTn vs no cTn elevation, p = NE - MI vs no cTn elevation, p = 0.174
Cardiogenic shock	2 (5.4)	0 (0)	0 (0)	0.162	- MI vs cTn, p = 1.000 - cTn vs no cTn elevation, p = NE - MI vs no cTn elevation, p = 0.174

cTn = cardiac troponin; MI = myocardial infarction; NE = not estimable; ^1^p value indicates a three-way comparison; ^2^p value indicates a two-way comparison; note that two patients had more than one MI, so the totals exceeds 100 per cent.

We report the use of antithrombotic agents and cardiac medications in Table 5. Patients with MI were more likely to receive antiplatelet medications, therapeutic dose heparin and long-acting nitrates compared to patients without cTn elevation.

**Table 5:** Cardiac medications

	MI, N (%) (*N *= 37)	Elevated cTn, N (%) (*N *= 15)	No cTn elevation, N (%) (*N *= 51)	p value^1^	p value^2^
Antiplatelet agents	26 (70.3)	7 (46.7)	16 (31.4)	0.001	- MI vs cTn, p = 0.126 - cTn vs no cTn elevation, p = 0.358 - MI vs no cTn elevation, p < 0.0001
Anticoagulant UFH	7 (19.4)	0 (0)	0 (0)	0.001	- MI vs cTn, p = 0.090 - cTn vs no cTn elevation, p = NE - MI vs no cTn elevation, p = 0.001
Anticoagulant LMWH	3 (8.3)	0 (0)	0 (0)	0.064	- MI vs cTn, p = 0.546 - cTn vs no cTn elevation, p = NE - MI vs no cTn elevation, p = 0.072
Anticoagulant Warfarin	8 (22.2)	3 (20.0)	4 (8.0)	0.156	- MI vs cTn, p = 1.000 - cTn vs no cTn elevation, p = 0.338 - MI vs no cTn elevation, p = 0.112
Long acting nitrates	19 (51.4)	5 (33.3)	6 (12.0)	<0.0001	- MI vs cTn, p = 0.358 - cTn vs no cTn elevation, p = 0.109 - MI vs no cTn elevation, p < 0.0001
Statin	19 (51.4)	7 (46.7)	18 (35.3)	0.306	- MI vs cTn, p = 1.000 - cTn vs no cTn elevation, p = 0.547 - MI vs no cTn elevation, p = 0.189
ACE inhibitors/ARBs	11 (29.7)	5 (33.3)	7 (14.0)	0.123	- MI vs cTn, p = 1.000 - cTn vs no cTn elevation, p = 0.128 - MI vs no cTn elevation, p = 0.108
Beta-blockers	25 (67.6)	5 (33.3)	20 (39.2)	0.014	- MI vs cTn, p = 0.032 - cTn vs no cTn elevation, p = 0.769 - MI vs no cTn elevation, p = 0.010
Calcium channel blocker	13 (35.1)	4 (26.7)	11 (21.6)	0.369	- MI vs cTn, p = 0.747 - cTn vs no cTn elevation, p = 0.731 - MI vs no cTn elevation, p = 0.225
Diuretic	25 (67.6)	9 (60.0)	17 (33.3)	0.004	- MI vs cTn, p = 0.749 - cTn vs no cTn elevation, p = 0.078 - MI vs no cTn elevation, p = 0.002
Digoxin	8 (22.2)	0 (0)	2 (3.9)	0.007	- MI vs cTn, p = 0.087 - cTn vs no cTn elevation, p = 0.594 - MI vs no cTn elevation, p = 0.014

ARBs = angiotensin receptor blockers; cTn = cardiac troponin; IV = intravenous; LMWH = low molecular weight heparin; MI = myocardial infarction; NE = not estimable; ^1^p value indicates a three-way comparison;^2^p value indicates a two-way comparison.

Based on clinical presentation, 18 patients (17.5 per cent) were diagnosed by the ICU team as having MI, but on adjudication, four of these patients did not meet criteria for MI (three patients did not have cTn elevation and the ECG of one patient was not adjudicated as meeting ESC/ACC criteria for MI). Of these 14 patients with MI detected clinically, 12 were NSTEMI and two were STEMI. Screening cTn and ECGs therefore identified 23 patients with MI who were 'missed' by routine clinical practice (reflecting 62.2 per cent of MIs ultimately diagnosed). Of these 23 patients with MI detected by screening, 22 were NSTEMI and one was STEMI. The outcome of patients with MI diagnosed clinically compared with MIs detected through screening is shown in Table 6. Of the 18 patients with MI diagnosed clinically by the ICU team, seven (38.9 per cent) died in the ICU and a further two died while in hospital (total hospital mortality of nine patients, representing 50 per cent mortality rate); nine patients (50 per cent) with clinically diagnosed MI were ultimately discharged from hospital. In comparison, of the 23 patients with MI diagnosed by screening, eight (34.8 per cent) died in the ICU and there were no further deaths while in hospital (total hospital mortality of eight patients, representing a 35 per cent mortality rate); 15 patients (65.2 per cent) with MI diagnosed by screening were ultimately discharged from hospital. Patients with MI diagnosed clinically were similar to patients with MI detected by screening alone with respect to ICU and hospital length of stay, and ICU and hospital mortality.

**Table 6:** Outcomes of patients with MI diagnosed clinically vs. MI diagnosed by screening

	MI diagnosed by ICU team (*N *= 18)	MI diagnosed by screening only (*N *= 23)	p value^1^
ICU mortality, N (%)	7 (38.9)	8 (34.8)	1.000^2^
Hospital mortality, N (%)	9 (50)	8 (34.8)	0.358^2^
ICU length of stay (mean days ± SD)	12.1 ± 16.4	8.5 ± 10.1	0.580^3^
Mean hospital length of stay (mean days ± SD)	33.8 ± 34.6	23.5 ± 25	0.287^3^

ICU = intensive care unit; MI = myocardial infarction; SD = standard deviation; ^1^p value indicates a two-way comparison; ^2^Chi-Square test; ^3^Mann-Whitney test.

## Discussion

In this observational screening study, 36 per cent of patients admitted to the ICU met diagnostic criteria for MI and 15 per cent had isolated cTn elevation during their ICU stay. Patients with MI had a longer ICU stay and increased hospital mortality compared with patients without cTn elevation; patients with MI also had a longer duration of mechanical ventilation and increased ICU mortality compared with those with and without cTn elevation. We documented that elevated cTn levels are associated with hospital mortality, even after adjusting for other important potential confounders. Patients with MI developed pulmonary oedema more frequently than those without MI, and were more likely to receive anti-ischaemic, antiplatelet and heparin therapy. Use of screening investigations detected more MIs than clinical diagnosis alone; however, the ICU and hospital length of stay and ICU and hospital mortality were similar for patients whose MIs were diagnosed by the ICU team and by screening.

This study is novel in that we performed both serial screening cTn measurements and ECG recordings on all patients admitted to the ICU. Screening investigations are relevant in this population since ICU patients frequently receive analgesic and sedative agents that can blunt pain and impair consciousness; moreover, they are commonly intubated and cannot communicate ischaemic symptoms that would typically initiate cardiac investigations. Thus, MI in the ICU setting is frequently undiagnosed. We previously audited the cTn ordering practice in this ICU without a screening protocol [2]. We found that 81 per cent of patients admitted to the ICU had at least 1 cTn measurement and 1 ECG; of these patients, 47 per cent had at least 1 elevated cTn level and 26 per cent met diagnostic criteria for MI by similar adjudication criteria as in the current study (elevated cTn and ischaemic ECG changes). The higher frequency of MI in the current study (36 per cent) is likely attributable to the screening process. In our previous study documenting cTn elevations noted by the ICU team, we also found that patients with MI compared with those without had significantly higher ICU and hospital mortality. Including MI identified by screening in the current study, we similarly found that patients with MI had significantly higher mortality in the ICU and hospital compared to patients without cTn elevation. Although subgroup analyses were not performed due to the relatively small number of patients with MI, there is a suggestion that patients at risk for MI more commonly had a history of hypertension, diabetes mellitus, prior myocardial infarction or congestive heart failure (Table 1).

Elevated cTn appears to be an adverse prognostic marker among critically ill patients. We found that elevated cTn was associated with increased hospital mortality (OR 27.3, 95 per cent CI 1.7 – 449.4). In a study of thoracic surgery and vascular surgery ICU patients, limited cTnT and ECG screening was performed but no difference in mortality rates was found between patients with and without cTn elevation [8]. In another study, ICU patients admitted with non-cardiac diagnoses who had an elevated cTn were found to have a four-fold higher mortality (22.4 vs. 5.2 per cent, p < 0.018) than patients without cTn elevation [5]. Among surgical ICU patients, moderate elevations in cTnI were associated with increased mortality and longer hospital and ICU length of stay compared to patients with normal cTn levels [9]. In a meta-analysis of 23 observational studies of critically ill patients, elevated cTn was associated with an adjusted OR for death of 2.5 (95 per cent CI 1.9–3.4) [7]. In unadjusted analyses with significant heterogeneity of results, elevated cTn was associated with an increased mean length of ICU stay of three days (95 per cent CI 0.98–5.05).

Since cTn is only released from damaged myocardial cells, elevated levels represent myocardial damage, which can plausibly increase the risk of death regardless of the mechanism. It is possible that because the aetiology of cTn elevation is variable in critically ill patients, the association with mortality and other adverse outcomes may also vary, and may be difficult to detect, thus requiring a larger sample size to estimate the true consequences of elevated cTn. Another issue influencing analysis of the impact of elevated cTn levels is that ECG interpretation, often done concurrently in practice, may have only moderate inter and intra-rater reliability in the ICU setting, which improves with knowledge of the cTn levels [10].

Recognising critically ill patients who have MI (versus an elevated cTn alone) may be important since these patients may benefit from antithrombotic and anti-ischaemic medications that have been shown to benefit non-critically ill patients. Whether these agents improve the outcome of patients with cTn elevation alone in the absence of ECG changes is unknown. Furthermore, the safety of these medications in critically ill patients with cTn elevation and ECG changes should be further explored since certain agents used widely in the ICU (such as beta-agonists) may be incompatible with cardiac medications (such as beta-blockers).

Strengths of the current study include the use of systematic screening investigations to examine cTn and ECG abnormalities that were not detected by the ICU team. The diagnosis of MI was made independently and in duplicate, blinded to the patient's clinical information. Furthermore, the cTn and ECG abnormalities documented during screening procedures were unavailable to the ICU team so they could not influence practice. We used multivariable analysis to examine the association between elevated cTn and MI with mortality. Limitations to the current study include the sample size and relatively small number of patients in each subgroup. Although we examined medication use among the subgroups, we cannot make inferences from this cohort study about the effectiveness of these medications. Lastly, the results of this study may not be applicable to other settings with a different patient case-mix, such as trauma units.

The mechanism for cTn elevation may differ in critically ill patients compared with conscious non-ICU patients presenting with chest discomfort and cTn elevation, but ultimately both cases result in myocardial cell necrosis. As per the current consensus definition of MI whereby any amount of myocardial necrosis reflecting any degree of cTn elevation can be considered an infarction [6], we used the term 'myocardial infarction' without implying the possible mechanism. Further research is needed to better understand mechanisms for cTn elevations in critically ill patients.

## Conclusion

Elevated cTn levels are common in critically ill patients when assessed by screening, and appear to have an important prognostic association with increased hospital mortality. We found that approximately one-third of patients also had ischaemic ECG changes in addition to elevated cTn levels, suggesting MI. Further research is needed to evaluate whether screening for MI and subsequent treatment of these patients reduces mortality.

## Key messages

Systematic screening using cardiac troponin (cTn) measurements and 12-lead electrocardiograms (ECGs) in consecutive critically ill patients admitted to a general medical-surgical intensive care unit (ICU) detected elevated cTn and ischaemic ECG changes supporting a diagnosis of myocardial infarction (MI) in 36 per cent of patients.

Patients meeting diagnostic criteria for MI had a longer ICU stay and increased hospital mortality compared with patients without cTn elevation.

Elevated cTn is predictive of increased hospital mortality.

Use of screening cTn measurements and 12-lead ECGs detected MI at a higher rate than clinical diagnosis alone.

Patients with MI detected by screening had similar outcomes compared with patients with MI diagnosed clinically by the ICU team.

## Abbreviations

APACHE, Acute Physiology and Chronic Health Evaluation; CI = confidence interval; cTn = cardiac troponin; cTnT = cardiac troponin T; ECG = electrocardiogram; ESC/ACC = European Society of Cardiology/American College of Cardiology; ICU = intensive care unit; IQR = interquartile range; MI = myocardial infarction; NSTEMI = non-ST elevation myocardial infarction; OR = odds ratio; STEMI = ST elevation myocardial infarction.

## Competing interests

The authors declare that they have no competing interests.

## Authors' contributions

DC: Conception and design, Collection and assembly of data, Statistical expertise, Drafting of the article, Administrative, technical, or logistic support, Final approval of the article; WL: Conception and design, Statistical expertise, Drafting of the article, Final approval of the article; PJD: Conception and design, Statistical expertise, Drafting of the article, Administrative, technical, or logistic support, Final approval of the article; MC: Conception and design, Drafting of the article, Administrative, technical, or logistic support, Final approval of the article; PH: Collection and assembly of data, Critical revision of the article for important intellectual content, Administrative, technical, or logistic support, Final approval of the article; AT: Collection and assembly of data, Critical revision of the article for important intellectual content, Administrative, technical, or logistic support, Final approval of the article; EM: Collection and assembly of data, Critical revision of the article for important intellectual content, Administrative, technical, or logistic support, Final approval of the article; FC: Collection and assembly of data, Critical revision of the article for important intellectual content, Administrative, technical, or logistic support, Final approval of the article; IQ: Collection and assembly of data, Critical revision of the article for important intellectual content, Administrative, technical, or logistic support, Final approval of the article; IT: Statistical expertise, Final approval of the article; HS: Statistical expertise, Critical revision of the article for important intellectual content, Administrative, technical, or logistic support, Final approval of the article

## References

[B1] King DA, Codish S, Novack V, Barski L, Almog Y (2005). The role of cardiac troponin I as a prognosticator in critically ill medical patients: a prospective observational cohort study. Crit Care.

[B2] Lim W, Qushmaq I, Cook DJ, Crowther MA, Heels-Ansdell D, Devereaux PJ (2005). Elevated troponin and myocardial infarction in the intensive care unit: A prospective study. Crit Care.

[B3] Jeremias A, Gibson CM (2005). Narrative review: Alternative causes for elevated cardiac troponin levels with acute coronary syndromes are excluded. Ann Intern Med.

[B4] Roongsritong C, Warraich I, Bradley C (2004). Common causes of troponin elevations in the absence of acute myocardial infarction: incidence and clinical significance. Chest.

[B5] Ammann P, Maggiorini M, Bertel O, Haenseler E, Joller-Jemelka HI, Oechslin E (2003). Troponin as a risk factor for mortality in critically ill patients without acute coronary syndromes. J Am Coll Cardiol.

[B6] Alpert JS, Thygesen K, Antman E, Bassand JP (2000). Myocardial infarction redefined – a consensus document of The Joint European Society of Cardiology/American College of Cardiology Committee for the redefinition of myocardial infarction. J Am Coll Cardiol.

[B7] Lim W, Qushmaq I, Devereaux PJ, Heels-Ansdell D, Lauzier F, Ismaila AS (2006). Elevated cardiac troponin measurements in critically ill patients. Arch Intern Med.

[B8] Klein Gunnewiek JMT, van de Leur JJJ (2003). Elevated troponin T concentrations in critically ill patients. Intensive Care Medicine.

[B9] Relos RP, Hasinoff IK, Beilman GJ (2003). Moderately elevated serum troponin concentrations are associated with increased morbidity and mortality rates in surgical intensive care unit patients. Crit Care Med.

[B10] Lim W, Qushmaq I, Cook DJ, Devereaux PJ, Heels-Ansdell D, Crowther MA (2006). The reliability of electrocardiogram interpretation in critically ill patients. Crit Care Med.

